# Characterization of *Verbesina encelioides* (Asteroideae, Asteraceae) Chloroplast Genome and Phylogenetic Insights

**DOI:** 10.1002/ece3.72897

**Published:** 2026-01-02

**Authors:** Rushan Yan, Abdullah, Jingjing Jia, Madiha Islam, Hui Li, Mengyang Liu, Bartholomew Yir‐erong, Xiaoxuan Tian

**Affiliations:** ^1^ State Key Laboratory of Chinese Medicine Modernization Tianjin University of Traditional Chinese Medicine Tianjin China; ^2^ Haihe Laboratory of Modern Chinese Medicine Tianjin China; ^3^ Department of Genetics Hazara University Manshera Pakistan; ^4^ State Key Laboratory of Component‐Based Chinese Medicine Tianjin University of Traditional Chinese Medicine Tianjin China; ^5^ Institute of Traditional and Alternative Medicine (ITAM), University of Health and Allied Sciences (UHAS) Ho Ghana

**Keywords:** adaptive evolution, Asteraceae, Heliantheae, phylogenetics, *Verbesina*

## Abstract

*Verbesina encelioides* (Cav.) Benth. & Hook.f. ex A.Gray (Asteroideae, Asteraceae) is a widespread annual herb native to southwestern North America that has naturalized globally. Here, we report the first de novo assembly and comprehensive annotation of the complete chloroplast (cp) genome of *V. encelioides* , generated using Illumina NovaSeq sequencing. The circular genome is 152,213 bp and exhibits the characteristic quadripartite structure, comprising a large single‐copy (83,911 bp) region, a small single‐copy (18,248 bp) region, and two inverted repeat regions (25,027 bp each). The genome encodes 112 unique genes, including 79 protein‐coding genes, 29 tRNAs, and four rRNAs, with 16 genes duplicated in the IRs. Comparative analysis with *Verbesina alternifolia* revealed high structural conservation regarding gene content and arrangement, codon usage, amino acid frequency, and simple sequence repeats. Codon usage showed bias toward A/T‐ending codons (RSCU > 1), whereas leucine was the most abundant amino acid, and cysteine was the least frequent. Simple sequence repeat analysis predominantly identified A/T‐rich mononucleotide repeats. Nucleotide diversity analysis highlighted several variable regions—including *trnD‐trnY*, *atpA‐trnR*, *rpl32‐trnL*, *ccsA‐ndhD*, and *trnL‐ccsA*—which may serve as molecular markers. Analysis of adaptive evolution in *Verbesina* species and related genera identified codons under positive selection in 19 chloroplast genes: *atpB*, *ccsA*, *clpP*, *ndhD*, *ndhI*, *psaB*, *psbB*, *rpl14*, *rpoB*, *rpoC1*, *rps8*, *ycf3*, *accD*, *matK*, *rbcL*, *ndhF*, *rpoC2*, *ycf2*, and *ycf1*. Maximum likelihood phylogenetic analysis placed *Verbesina* within the tribe Heliantheae. This complete cp genome provides a valuable genetic resource for phylogenetic studies, DNA barcoding, and population genetics of *Verbesina* and related Asteraceae taxa.

## Graphical abstract

In this study, we report the first de novo assembled chloroplast genome of *Verbesina encelioides*, a member of the tribe Heliantheae within the subfamily Asteroideae of Asteraceae.

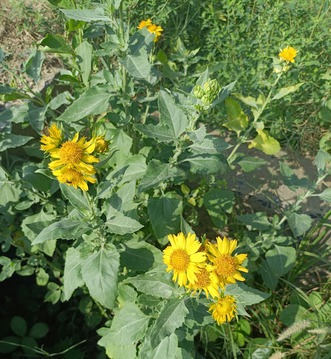

## 1 Introduction

Asteraceae, one of the largest and most ecologically successful angiosperm families, comprises approximately 1620 genera and 34,000 species distributed across 16–17 subfamilies (Zhang, Yang, et al. 2024; WFO 2025). Members of Asteraceae occur worldwide, including Antarctica, and occupy diverse habitats. They display remarkable morphological and ecological plasticity, comprising annual and perennial herbs, shrubs, trees, vines, and succulents (Funk 2009; Susanna and Baldwin 2020; Roeble et al. 2024).

The genus *Verbesina* L., within the subtribe Verbesininae of the tribe Heliantheae, subfamily Asteroideae, is endemic to the Americas and comprises 351 species (POWO 2025). To date, only one species in the genus, *Verbesina alternifolia* (L.) Britton ex Kearney, has a published chloroplast (cp) genome (Tomasello et al. 2024). *Verbesina encelioides* (Cav.) Benth. & Hook.f. ex A.Gray, native to southwestern North America, has naturalized in disturbed areas globally. This annual herb thrives in temperate to subtropical biomes and is well adapted to arid and sandy soils (Gorja and Bandla 2024). Owing to its aggressive spread, strong ecological competitiveness, and invasive behavior in many regions, *V. encelioides* has become a focal species in studies of biological invasion, abiotic‐stress adaptation, and ecological risk assessment (Menghani 2008; Singh 2010; Mehal et al. 2023). However, the cp genome of this species remains unavailable, limiting our understanding of its dispersal mechanisms and evolutionary history. Characterizing the complete cp genome would enable phylogeographic analyses, facilitate the development of robust molecular markers, and support invasive species management strategies. Moreover, *V. encelioides* exhibits important pharmacological activities and is widely used in traditional medicine (Kataria et al. 2025), making genomic resources valuable for both ecological and medicinal research applications.

The cp genome typically exhibits a conserved quadripartite structure, comprising a large single‐copy (LSC) region, a small single‐copy (SSC) region, and two inverted repeats (IRa and IRb) (Palmer 1985; Daniell et al. 2016). Owing to its moderate polymorphism and predominantly maternal inheritance in angiosperms, the cp genome serves as a valuable resource for phylogenetic reconstruction, population genetics, DNA barcoding, and conservation studies (Daniell et al. 2016; Zhang, Huang, et al. 2024; Zeng et al. 2025). Several mutations have been reported in cp genomes, including substitutions, insertions–deletions, inversions, and contraction or expansion of inverted repeats, which can alter gene content (Daniell et al. 2016; Zhang, Huang, et al. 2024; Abdullah, Haram, et al. 2025; Yan et al. 2025; Abdullah, Li, et al. 2025).

To expand genomic resources for *Verbesina*, we sequenced and de novo assembled the *V. encelioides* cp genome to characterize its genetic features, conduct comparative analyses with *V. alternifolia* , identify polymorphic loci with potential phylogenetic utility, assess signatures of molecular evolution, and reconstruct its phylogenetic relationships within the tribe. This study provides the first complete cp genomic resource for *V. encelioides* , filling a critical data gap and offering a molecular foundation for future evolutionary, ecological, and invasion biology research on this globally expanding species.

## 2 Materials and Methods

### 2.1 Sample Collection, DNA Extraction, and Sequencing

**FIGURE 1:**
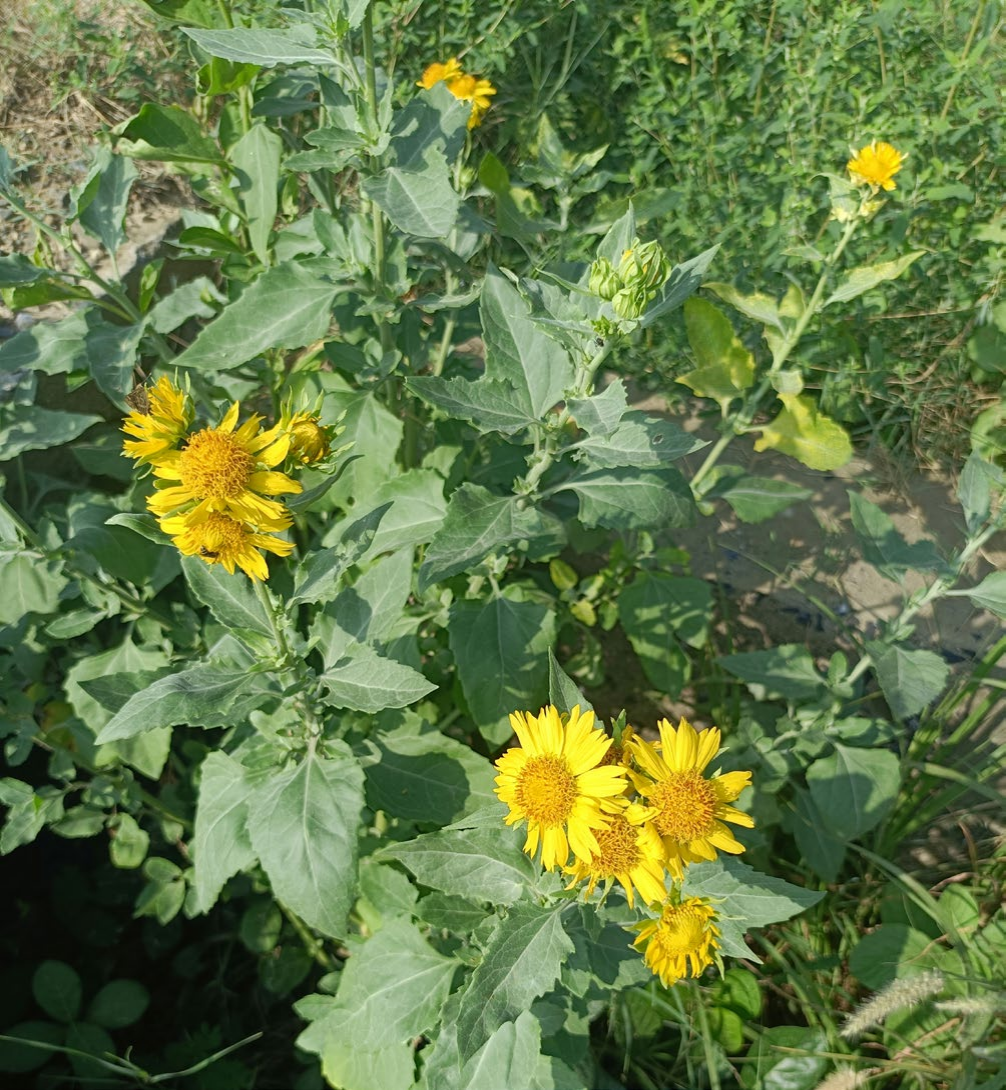
*Verbesina encelioides* in its natural habitat. The photograph, taken by Dr. Abdullah, highlights several inflorescences of *V. encelioides* . Given the dense surrounding vegetation and the coexistence of other plant species, the image emphasizes clearly distinguishable flowers to facilitate accurate identification.

*V. encelioides* was collected from District Nowshera, Khyber Pakhtunkhwa Province, Pakistan (34°04′38.68″ N, 71°90′97.17″ E) and identified by Dr. Abdul Majid, Assistant Professor at Hazara University, Mansehra. The voucher specimen was submitted to the herbarium of Hazara University, Mansehra, under accession number HUP‐17585. Permission was not required from the national or local authorities for plant collection and utilization in research. A photograph of the plant is provided in Figure 1. Leaves were dried using silica gel for DNA extraction. DNA was extracted from 30 mg of dried leaf tissue using the Plant Genomic DNA Kit (TIANGEN BIOTECH, Beijing, China) with the following modifications: (a) tissue was homogenized in 1.5 mL of GP1 buffer, and the resulting lysate was divided into two 1.5‐mL microcentrifuge tubes for parallel processing; (b) the incubation at 65°C was extended to 60 min; (c) supernatants from both tubes were combined and loaded onto a single DNA‐binding column (CB3); and (d) final elution was performed with 95 μL of elution buffer after a 40‐min incubation at room temperature.

DNA quality and concentration were evaluated using an Agilent 5400 System (Agilent Technologies), which confirmed high‐molecular‐weight DNA at 19.74 ng/μL (total yield ≈ 1.8 μg). A library with a 350‐bp insert size was prepared, and paired‐end sequencing (2 × 150 bp) was performed on an Illumina NovaSeq 6000 platform at Novogene (Tianjin, China).

### 2.2 Chloroplast Genome Assembly and Annotation

We assessed raw read quality using Fastp v1.0.1 (Chen 2025). Reads containing > 10% ambiguous nucleotides (N) or > 50% bases with quality score (Q) ≤ 5 were filtered out. The resulting clean reads, which had Q20 and Q30 scores of 99% and 96%, respectively, were used for de novo cp genome assembly using GetOrganelle v1.7.5^+^ (Jin et al. 2020) with k‐mer sizes of 21, 45, 65, 85, and 105.

Genome annotation was performed using GeSeq v2.03 (Tillich et al. 2017), while transfer RNA genes were further verified using ARAGORN v1.2.38 (Laslett and Canback 2004) and tRNAscan‐SE v2.0.7 (Chan and Lowe 2019). Start and stop codons of protein‐coding sequences (CDS) were manually verified and corrected in Geneious Prime 2025, based on comparisons with *Verbesina alternifolia* (PP_639077), *Eclipta alba* (NC_039774), and *Silphium integrifolium* (NC_068130).

### 2.3 Comparative Genomics, Amino Acid Frequency, and Codon Usage Analysis

The cp genomes of *V. alternifolia* and *V. encelioides* were compared using Geneious Prime 2025 and the mVISTA tool (Frazer et al. 2004) in Shuffle‐LAGAN mode. Amino acid frequency and relative synonymous codon usage (RSCU) were analyzed using custom Python scripts (Abdullah, Fatima, et al. 2025).

### 2.4 Analysis of Nucleotide Substitutions and Microsatellites

To analyze nucleotide substitutions, the LSC, SSC, and IR regions of *V. alternifolia* and *V. encelioides* were extracted and aligned pairwise. Transition and transversion substitutions were identified, and their ratios were calculated using a custom Python script (Script 1). Simple sequence repeats (SSRs) were identified using MISA‐web (https://webblast.ipk‐gatersleben.de/misa/) (Beier et al. 2017). Search parameters were set to a minimum of 10 repeat units for mononucleotides, 5 for dinucleotides, 4 for trinucleotides, and 3 for tetranucleotides, pentanucleotides, and hexanucleotides.

### 2.5 Analysis of Adaptive Evolution

To evaluate signatures of episodic diversifying selection, HyPhy v2.5.7 (Pond et al. 2005) was used to implement the Mixed Effects Model of Evolution (MEME) (Murrell et al. 2012). Codon‐based alignments were generated separately for each protein‐coding gene using MUSCLE v5 (Edgar 2022), and terminal stop codons were removed in Geneious prior to analysis. A total of 15 complete chloroplast genomes representing 15 closely related Asteraceae species were included (Table S1). For each gene, a maximum‐likelihood phylogeny was reconstructed using FastTree v2 (Price et al. 2009) with default settings, as required for MEME to infer branch‐specific variation in selective pressures.

### 2.6 Analysis of Nucleotide Polymorphism and Phylogenetic

Nucleotide diversity (Pi) for all cp genome regions, including introns, CDS, and intergenic spacers (IGS) regions, was calculated using CPStools (L. Huang et al. 2024).

We retrieved 38 species from the National Centre for Biotechnology Information (NCBI) belonging to distinct genera of tribe Heliantheae and aligned them with *Verbesina* species, including *Blumea aromatica* (NC_069835) from tribe Inuleae as the outgroup, using MAFFT (Nakamura et al. 2018). The phylogenetic tree was reconstructed from whole‐genome alignments using the maximum likelihood (ML) method implemented in IQ‐TREE v3.0.1 (Wong et al. 2025) with automated model selection (ModelFinder), SH‐aLRT branch support, and 1000 ultrafast bootstrap replicates with the ‐‐bnni option, following a previous description (Abdullah, Haram, et al. 2025). The resulting trees were inferred under the BIC‐selected model (GTR + F) and visualized using the Interactive Tree of Life platform (iTOL) (Letunic and Bork 2024; http://itol.embl.de/).

## 3 Results and Discussion

### 3.1 Characterization of the Chloroplast Genome of *Verbesina encelioides*

**FIGURE 2:**
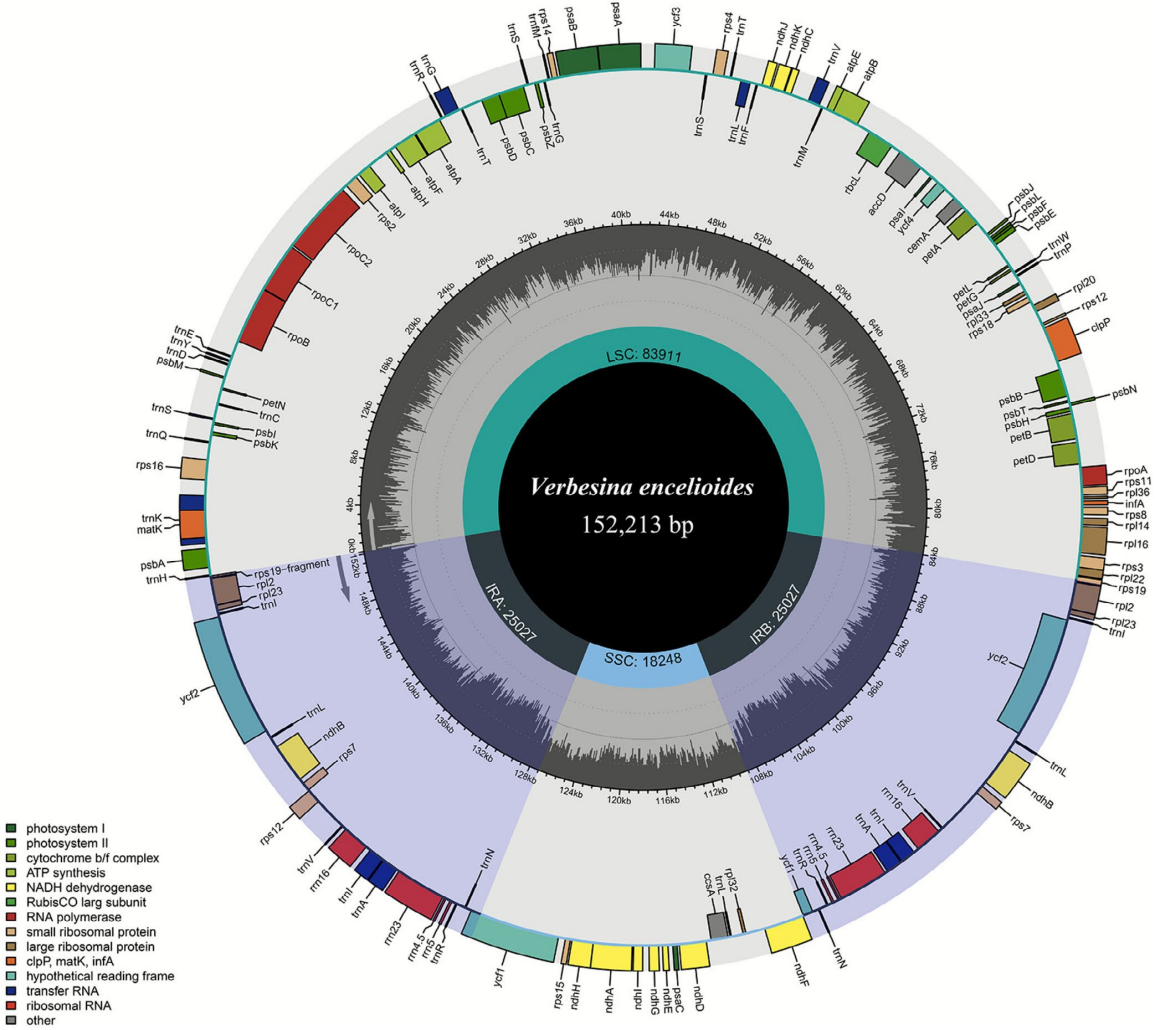
Circular map depicting the chloroplast genome of *V. encelioides* . Genes transcribed clockwise are shown inside the circle, whilst those transcribed anticlockwise are shown outside the circle. Genes are color‐coded based on function. Darker gray in the inner circle represents GC content throughout the genome.

Sequencing *V. encelioides* generated approximately 5.44 GB of data, yielding 7.8 million paired‐end clean reads (150 bp each). The cp genome was assembled de novo with an exceptionally high average coverage depth of 2281× from 2.3 million cp‐specific reads. The complete cp genome measured 152,213 bp and exhibited the typical quadripartite structure (Figure 2), comprising the LSC region (83,911 bp), the SSC region (18,248 bp), and two IR regions (IRa/IRb; 25,027 bp each). Such high coverage ensures reliable assembly and supports robust downstream comparative analyses. The quadripartite organization observed here is characteristic of most land plant cp genomes and reflects the evolutionary conservation of this structural arrangement across diverse angiosperm lineages (Abdullah, Haram, et al. 2025; Y. Huang et al. 2025; Xing et al. 2025).

The overall GC content was 37.7%, with marked regional variation: LSC (35.8%), IRs (43.1%), and SSC (31.5%). Among gene classes, GC content was highest in rRNA genes (55.2%), followed by tRNAs (53.1%) and protein‐coding sequences (CDS) (37.9%). Such GC variation likely reflects differential selective pressures, with higher GC content in IR regions contributing to structural stability and conservation. The genome showed high similarity in length and GC content to other *Verbesina* species (Table 1) and to previously reported species from the tribe Heliantheae and the subfamily Asteroideae (Abdullah et al. 2021; Mahai et al. 2024; Karimov et al. 2025; Xue et al. 2025). This GC profile aligns with patterns observed across Asteroideae in genera such as *Artemisia* and *Blumea* (Iram et al. 2019; Abdullah et al. 2021), suggesting tribe‐level evolutionary constraints on GC content that are conserved across diverse ecological contexts rather than being species‐specific adaptations.

**TABLE 1:** Comparative analysis of chloroplast genome of *Verbesina* species.

Characteristics	*V. alternifolia*	*V. encelioides*
Total size (bp)	152,050	152,213
LSC length (bp)	83,725	83,911
SSC length (bp)	18,245	18,248
IR length (bp)	25,040	25,027
Unique genes	112	112
Protein‐coding genes	79	79
tRNA genes	29	29
rRNA genes	4	4
Duplicate genes	16	16
**GC content (%)**		
Total	37.7	37.7
LSC	35.8	35.8
SSC	31.4	31.5
IR	43.1	43.1
CDS	38	37.9
rRNA	55.2	55.2
tRNA	53	53.1
All gene	39.5	39.5

Abbreviations: bp = base pair, GC = Guanine‐cytosine, lR = inverted repeat, LSC = large single‐copy, rRNA = ribosomal RNA, SSC = small single‐copy, tRNA = transfer RNA.

The cp genome contained 112 unique genes: 79 CDS genes, 29 transfer RNA (tRNA) genes, and four ribosomal RNA (rRNA) genes. Sixteen genes were duplicated within the IR regions, including five CDS genes (*ndhB*, *rpl2*, *rpl23*, *rps7*, and *ycf2*), four rRNA genes (*rrn16S*, *rrn23S*, *rrn4.5S*, and *rrn5S*), and seven tRNA genes (*trnA‐UGC, trnI‐GAU, trnL‐CAA, trnN‐GUU, trnR‐ACG, trnV‐GAC, trnI‐CAU*) (Table 2). These duplicated genes enhance genome stability by providing redundancy for essential functions.

**TABLE 2:** Chloroplast gene content and functional classification in *Verbesina* species.

Category for genes	Group of genes	Name of genes	Amount
Self‐replication	Large subunit of ribosome	*rpl2* ^*a*^ (2), *rpl14*, *rpl16* ^*a*^, *rpl20*, *rpl22*, *rpl23*(2), *rpl32, rpl33*, *rpl36*	11
	Small subunit of ribosome	*rps2*, *rps3*, *rps7*(2), *rps4*, *rps15*, *rps8*, *rps11*, *rps16* ^*a*^, *rps18*, *rps14*, *rps12* ^*a*^, *rps19*	13
	DNA dependent RNA polymerase	*rpoA*, *rpoB*, *rpoC1* ^*a*^, *rpoC2*	4
	rRNA genes	*rrn5*(2), *rrn16*(2), *rrn23*(2), *rrn4.5*(2)	8
	tRNA genes	*trnA‐UGC* ^*a*^(2), *trnC‐GCA*, *trnD‐GUC*, *trnE‐UUC*, *trnF‐GAA*, *trnG‐GCC*, *trnG‐UCC* ^*a*^, *trnH‐GUG*, *trnI‐CAU*(2), *trnI‐GAU* ^*a*^(2), *trnK‐UUU* ^*a*^, *trnL‐CAA*(2), *trnL‐UAA* ^*a*^, *trnL‐UAG*, *trnM‐CAU*, *trnN‐GUU*(2), *trnP‐UGG*, *trnQ‐UUG*, *trnR‐ACG*(2), *trnR‐UCU* ^*a*^, *trnS‐GCU*, *trnS‐GGA*, *trnS‐UGA*, *trnT‐UGU*, *trnV‐GAC*(2), *trnV‐UAC* ^*a*^, *trnW‐CCA*, *trnY‐GUA*, *trnfM‐CAU*	36
Photosynthesis	Photosystem I	*psaA*, *psaB*, *psaC*, *psaI*, *psaJ*	5
	Photosystem II	*psbA*, *psbB*, *psbC*, *psbD*, *psbE*, *psbF*, *psbH*, *psbI*, *psbJ*, *psbK*, *psbL*, *psbM*, *psbN*, *psbT*, *psbZ*	15
	NADPH dehydrogenase	*ndhA* ^*a*^, *ndhB* ^*a*^(2), *ndhC*, *ndhD*, *ndhE*, *ndhF*, *ndhG*, *ndhH*, *ndhI*, *ndhJ*, *ndhK*	12
	Cytochrome b/f complex	*petA*, *petB* ^*a*^, *petD* ^*a*^, *petG*, *petL*, *petN*	6
	Subunits of ATP synthase	*atpA*, *atpB*, *atpE*, *atpF* ^*a*^, *atpH*, *atpI*	6
	Photosystem I assembly proteins	*ycf3* ^*b*^, *ycf4*	2
	Large subunit of Rubisco	*rbcL*	1
Other genes	Protease	*clpP* ^*b*^	1
	Maturase	*matK*	1
	Envelop membrane protein	*cemA*	1
	Subunit of Acetyl‐CoA‐carboxylase	*accD*	1
	C‐type cytochrome synthesis gene	*ccsA*	1
	Translation initiation factor	*infA*	1
	Conserved open reading frames	*ycf1*, *ycf2*(2)	3

*Note:* Gene^a^, Gene with one intron; Gene^b^, Gene with two introns; Gene (2): Number of copies of multi‐copy genes.

**FIGURE 3:**
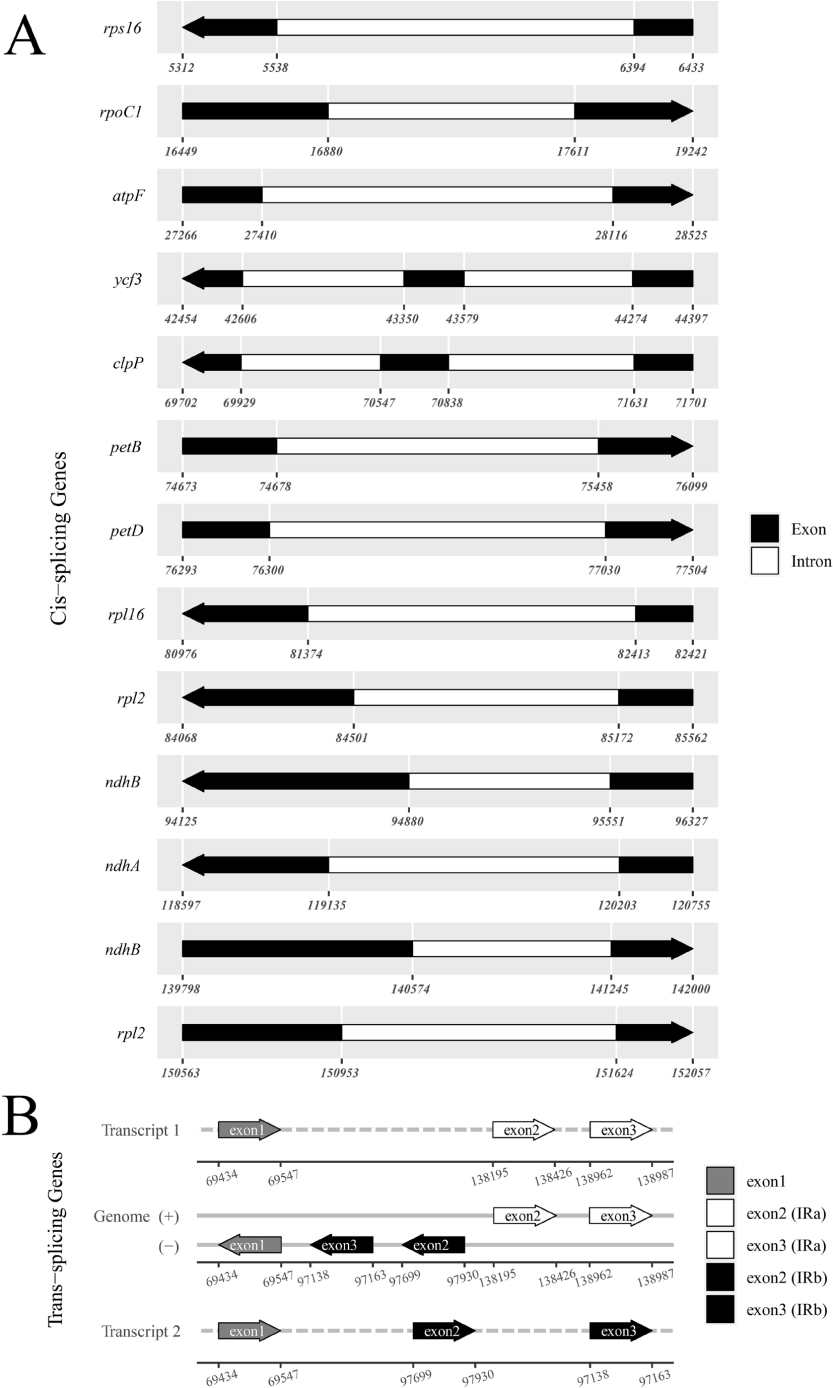
Gene structure map of cis‐ and trans‐spliced genes in the *V. encelioides* chloroplast genome. (A) Cis‐spliced genes. (B) Trans‐spliced genes.

Eighteen genes contained introns: 12 CDS (*atpF, petB, rps16, rps12, rpl2, ndhA, rpl16, ndhB, rpoC1, ycf3, petD, clpP*) and six tRNAs (*trnV‐UAC, trnK‐UUU, trnG‐UCC, trnI‐GAU, trnL‐UAA, trnA‐UGC*). Two CDS genes (*ycf3* and *clpP*) contained two introns each, whilst the remaining 10 had a single intron (Figure 3A). Notably, *rps12* was trans‐spliced, with exon 1 in the LSC region and exons 2 and 3 in the IR regions (Figure 3B). This trans‐splicing reflects a conserved mechanism requiring precise coordination during RNA processing. Gene content and intron distribution were consistent with previous reports for tribe Heliantheae and subfamily Asteroideae (Abdullah et al. 2021; Mahai et al. 2024; Karimov et al. 2025; Xue et al. 2025), indicating a highly conserved cp genome structure within this lineage. This conservation is maintained across both invasive and non‐invasive species, demonstrating that invasive properties have not driven structural rearrangements or alterations in gene content within the cp genome. This finding further supports the hypothesis that traits conferring invasiveness are likely governed by nuclear rather than chloroplast genes.

### 3.2 Comparative Genomics, Amino Acid Frequency, Codon Usage, and Simple Sequence Repeats Analysis

**FIGURE 4:**
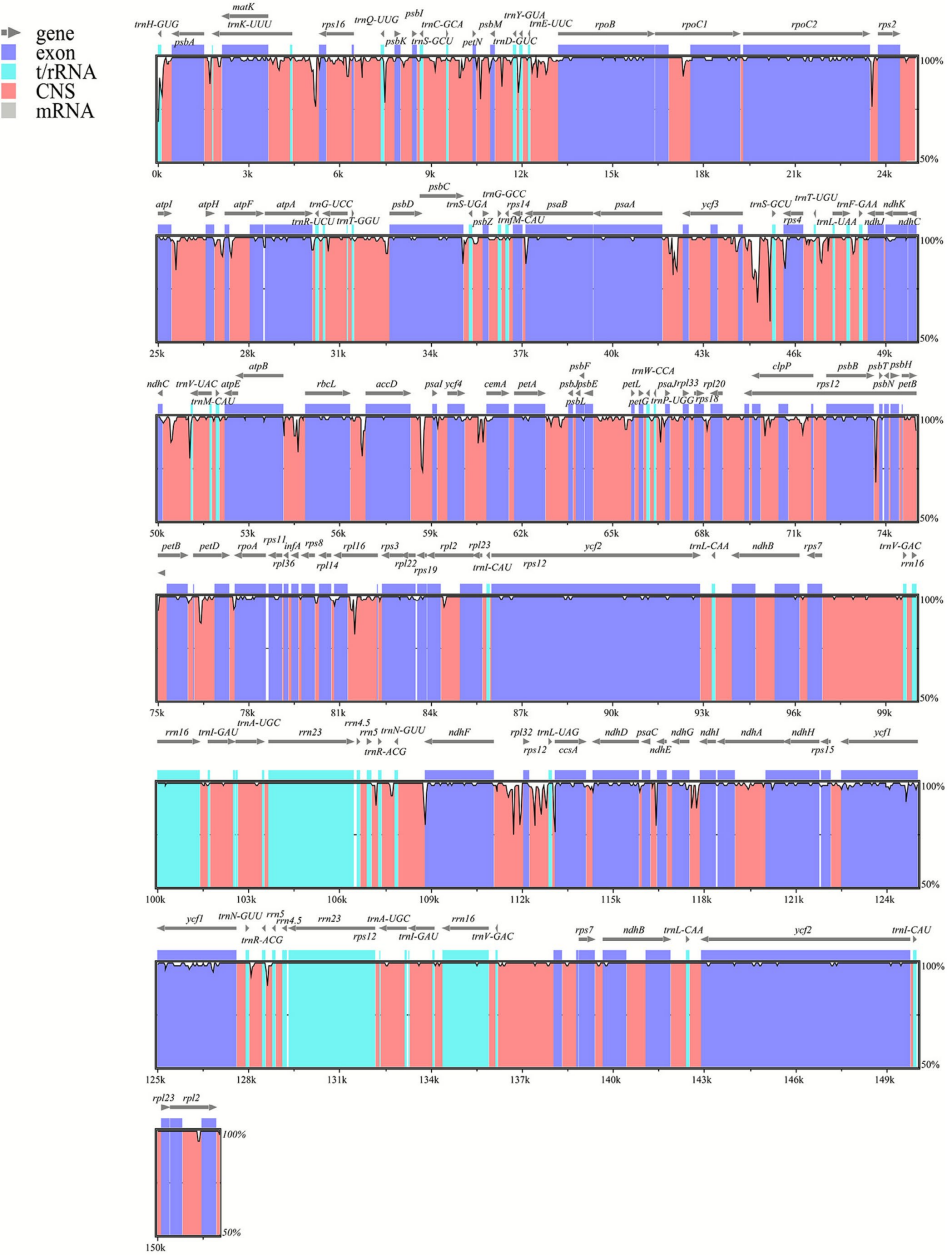
Global alignment of chloroplast genomes of *V. alternifolia* and *V. encelioides* . We use *V. alternifolia* as a reference. The *x*‐axis represents the coordinates in the chloroplast genome. The *y*‐axis shows the average percent identity of the aligned regions, ranging from 50% to 100%.

Comparative genomic analysis between *V. encelioides* and *V. alternifolia* revealed high conservation. Intergenic spacers and intron regions were more variable than coding sequences (Figure 4). Most CDS regions were highly conserved, whereas rRNA genes were virtually invariant, reflecting strong purifying selection on coding regions. The invariant nature of rRNA genes underscores their essential role in ribosome assembly and protein synthesis, where mutations are likely deleterious. Similar patterns have been reported in *Artemisia*, *Blumea*, and other Asteroideae taxa (Iram et al. 2019; Abdullah et al. 2021; Xing et al. 2025), as well as in dicot families such as Solanaceae, Phyllanthaceae, and Malvaceae (Abdullah, Mehmood, et al. 2020; Abdullah et al. 2021; Rehman et al. 2021). Within Heliantheae, this pattern suggests evolutionary pressures favoring stability in core photosynthetic and translational machinery despite ecological diversification. Notably, the absence of invasiveness‐specific positive selection in the cp genome (discussed in the adaptive evolution section) further supports the hypothesis that traits underlying the invasive success of *V. encelioides* are likely encoded in the nuclear genome rather than the chloroplast.

**FIGURE 5:**
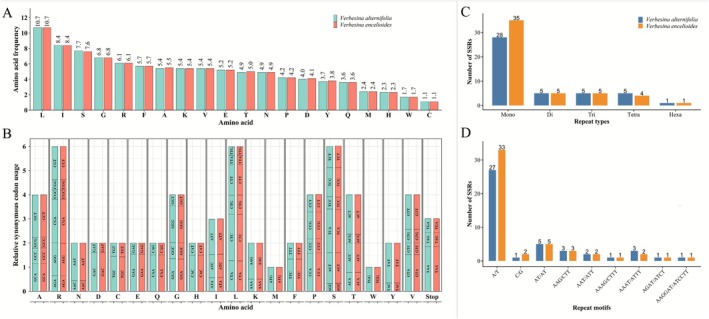
Analysis of amino acid frequency, codon usage, and simple sequence repeats (SSRs) in the chloroplast genomes of *V. alternifolia* and *V. encelioides* . (A) Amino acid frequency distribution, with amino acid types on the *x*‐axis and their encoded frequency on the *y*‐axis. (B) Relative synonymous codon usage (RSCU), where the *x*‐axis indicates amino acids and bar height represents the RSCU value for each species; codons are labeled inside bars. (C) Identified SSR types. (D) SSR motif compositions.

Amino acid frequency analysis showed leucine was the most abundant, whereas cysteine was the least encoded (Figure 5A). This reflects the hydrophobic nature of many cp proteins, especially those involved in photosynthesis, and the reducing environment of the cp stroma, which disfavors disulfide bond formation. Codon usage analysis revealed strong bias toward codons ending in A or T at the third position (RSCU > 1), whereas C‐ or G‐ending codons had RSCU < 1 (Figure 5B). This A/T‐ending codon bias is consistent with the high AT content of the cp genome and may influence tRNA abundance and translation efficiency. Similar patterns have been reported in other Asteroideae and plant lineages (Iram et al. 2019; Abdullah, Mehmood, et al. 2020; Abdullah et al. 2021; Rehman et al. 2021).

Simple sequence repeats (SSRs) were predominantly mononucleotide repeats (28–35 per genome), followed by di‐ and trinucleotide repeats (Figure 5C), whereas pentanucleotide repeats were absent and hexanucleotide repeats were extremely rare (1 locus) (Figure 5C,D). Most SSRs were composed of A/T‐rich motifs (27–33), fully reflecting the genome‐wide AT bias. These patterns are consistent with previous reports in *Blumea* and *Artemisia* (Iram et al. 2019; Abdullah et al. 2021). The prevalence of A/T‐rich SSRs reflects the genome's compositional bias and suggests that slipped‐strand mispairing occurs preferentially in AT‐rich regions. These SSR loci may serve as valuable molecular markers for population genetic studies and for tracing the invasion biogeography of *V. encelioides* across its expanding global range.

### 3.3 Nucleotide Substitutions

Pairwise comparison revealed more substitutions and a higher transition‐to‐transversion (Ts/Tv) ratio in the IR regions (1.5) compared to LSC (1.09) and SSC (1.08) (Table 3). The most frequent substitutions were A/G and C/T changes. Although reported Ts/Tv ratios vary in cp genomes, most studies indicate values ≤ 1 (Abdullah et al. 2019), with some reporting ratios > 1 (Cao et al. 2018; Abdullah, Henriquez, et al. 2020). Elevated Ts/Tv ratios in IR regions may reflect sequence context and secondary structure constraints that favor certain mutation types.

**TABLE 3:** Transition and transversion substitutions between *Verbesina* species.

Substitution type	LSC region	SSC region	IR region
A/G	115	45	7
G/T	73	24	3
A/C	63	20	3
C/T	109	43	5
C/G	27	5	2
A/T	42	32	0
Ts	224	88	12
Tv	205	81	8
Ts/Tv	1.09	1.08	1.5

### 3.4 Evaluation of Adaptive Evolution

MEME analysis identified 62 codon sites under episodic diversifying selection distributed across 19 chloroplast genes: *atpB*, *ccsA*, *clpP*, *ndhD*, *ndhI*, *psaB*, *psbB*, *rpl14*, *rpoB*, *rpoC1*, *rps8*, *ycf3*, *accD*, *matK*, *rbcL*, *ndhF*, *rpoC2*, *ycf2*, and *ycf1* (Table S1). These selected sites were highly concentrated in a few genes, with *ycf1* harboring the most (22 sites), followed by *ycf2* (7), *rpoC2* (7), and *ndhF* (6), while the remaining genes each contained one to three selected codons.

This enrichment in *ycf1* and *ycf2* is consistent with patterns observed across angiosperms. In *Zingiber*, for example, these genes exhibited 52 and 24 positively selected sites, respectively (Jiang et al. 2023), and similar selection signatures have been reported in *Erigeron* (Asteroideae, Asteraceae) (Abdullah, Rahmatulla, et al. 2025). The multiple selected codons detected in *ndhF*—a gene frequently implicated in adaptive radiations—mirror findings in *Erigeron* and other angiosperm lineages (Abdullah, Rahmatulla, et al. 2025; Corvalán et al. 2023). Concurrent positive selection in *ndhF* and *rpoC2*, which encodes a core subunit of the plastid‐encoded RNA polymerase involved in chloroplast transcription, has also been documented in *Oryza*, potentially reflecting adaptation to diverse ecological or light conditions (Gao et al. 2019).

The selection landscape in *V. encelioides* broadly parallels patterns documented throughout Asteraceae (Table S1), where adaptive evolution consistently targets large open reading frames (*ycf1*, *ycf2*) and genes central to photosynthesis or transcriptional regulation. The relatively high density of selected codons in *ycf1* and *ndhF* observed in *V. encelioides* is characteristic of these genes across the family rather than unique to this species. Thus, while these molecular signatures reflect conserved evolutionary pressures common to Asteraceae, they do not directly account for the species' invasive potential. This suggests that invasiveness in *V. encelioides* may instead be linked to genes in the nuclear genome.

### 3.5 Nucleotide Polymorphism and Phylogenetic Analysis

**FIGURE 6:**
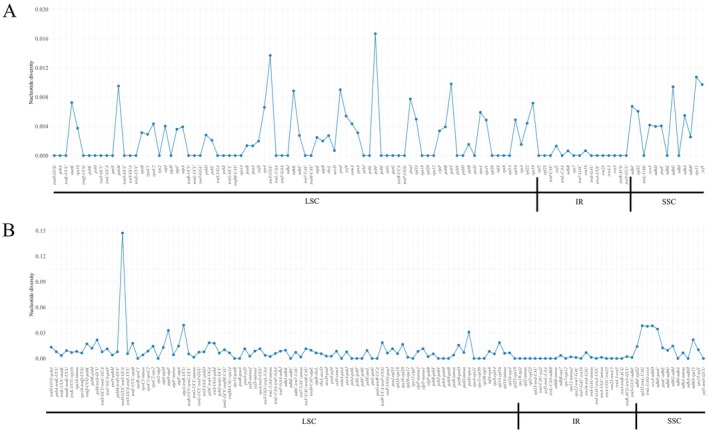
Nucleotide diversity of chloroplast genome regions. (A) Coding regions, including protein‐coding genes, tRNA genes, and rRNA genes. (B) Non‐coding regions, including intergenic spacer regions and intronic regions.

Nucleotide diversity (Pi) across CDS, tRNA, rRNA, intronic, and IGS regions was calculated from an alignment of the two *Verbesina* cp genomes. IGS regions exhibited the highest nucleotide diversity (Pi = 0.0093), followed by introns (Pi = 0.0043) and CDS (Pi = 0.0030) (Figure 6). Among CDS genes, *psbF*, *rps15*, *psbT*, *ycf1*, and *psbM* showed the highest variation (0.00952–0.01667). Highly polymorphic IGS regions included *trnD‐trnY*, *atpA‐trnR*, *rpl32‐trnL*, *ccsA‐ndhD*, and *trnL‐ccsA* (0.03774–0.14729) (Table 4). *trnL‐ccsA* had the highest missing data (17.8%), whereas *trnD‐trnY* showed the highest nucleotide polymorphism (0.77% missing).

**TABLE 4:** Identified suitable polymorphic loci based on comparative plastome analysis of *Verbesina species*.

Serial number	Region	Nucleotide diversity	Number of substitutions	Number of indels	Length (excluding indels)	Alignment length	Missing data (%)
**Protein coding region**							
1	*psbF*	0.01667	2	0	120	120	0
2	*rps15*	0.01075	3	0	279	279	0
3	*psbT*	0.0098	1	0	102	102	0
4	*ycf1*	0.00972	49	6	5043	5049	0.12
5	*psbM*	0.00952	1	0	105	105	0
**Intergenic region**							
6	*trnD‐trnY*	0.14729	19	1	129	130	0.77
7	*atpA‐trnR*	0.03906	5	4	128	132	3.0
8	*rpl32‐trnL*	0.03846	25	36	650	686	5.2
9	*ccsA‐ndhD*	0.03831	10	14	261	275	5.1
10	*trnL‐ccsA*	0.03774	4	23	106	129	17.8

Elevated diversity in IGS regions reflects reduced functional constraints, permitting neutral mutations to accumulate. Highly variable CDS genes such as *ycf1* and *psbM* may experience relaxed selective pressure or lineage‐specific adaptation. These polymorphic regions differ from those previously reported in *Blumea* and *Artemisia* (Iram et al. 2019; Abdullah et al. 2021), underscoring the value of species‐specific markers for phylogenetic resolution and DNA barcoding (Ahmed et al. 2013; X. Li et al. 2015; H. Li et al. 2025).

**FIGURE 7:**
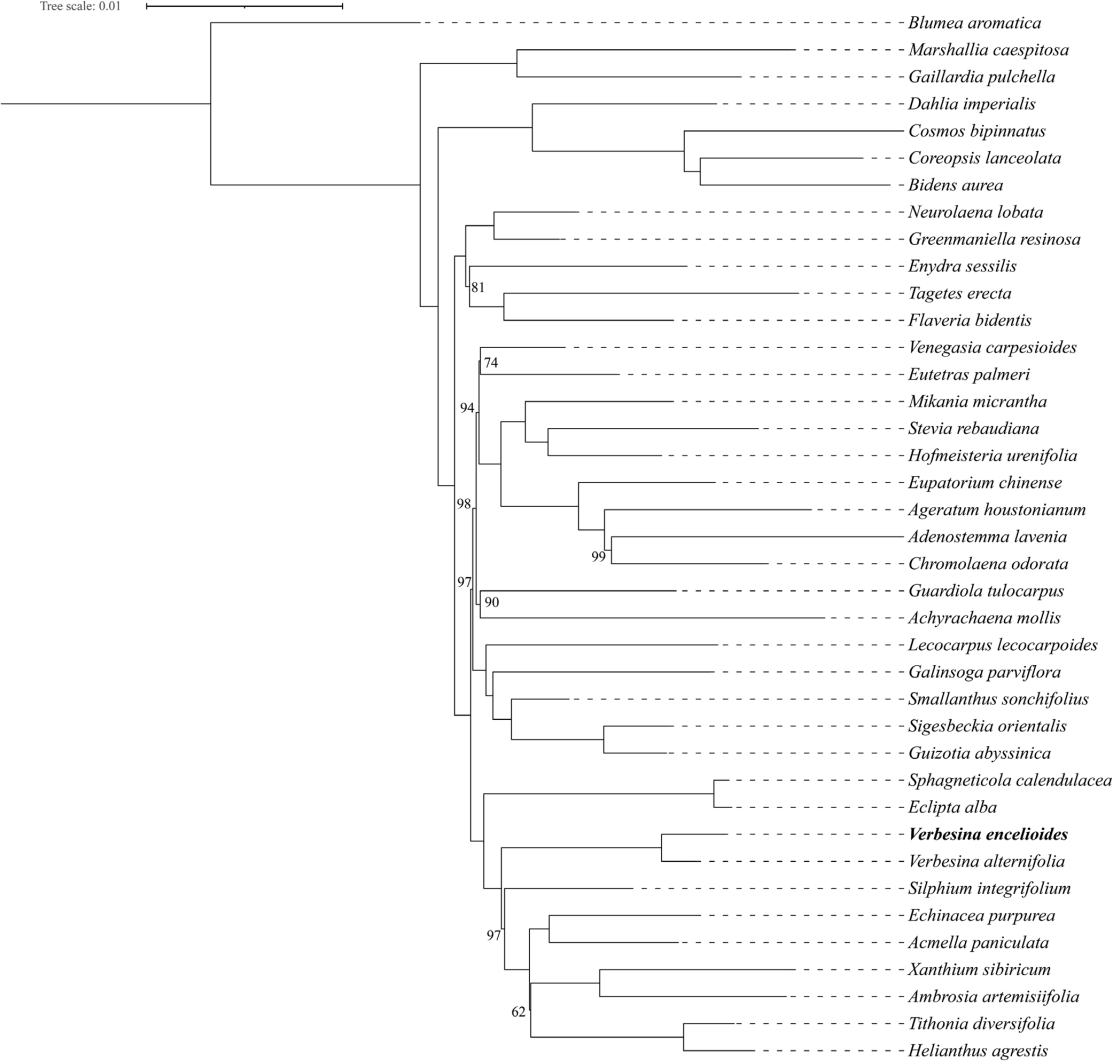
Maximum likelihood phylogenetic inference among Heliantheae. All nodes with bootstrapping values ≤ 99% are shown. The *V. encelioides* , which is sequenced in the current study, has been shown in bold.

The phylogenetic tree (Figure 7) robustly illustrates the evolutionary relationships among the major clades, with most key nodes receiving high bootstrap support (≥ 99%), indicating strong confidence in the topology. However, some nodes showed relatively lower support (bootstrap values of 62, 74, and 81). This reduced support may reflect rapid divergence during the evolutionary history of these lineages, limiting the resolution power of molecular markers, or may indicate reticulate evolution, a phenomenon previously documented in subfamily Asteroideae (Abdullah, Mehmood, et al. 2020). Nevertheless, the monophyly of major clades remains well‐supported and consistent with previous reports (Schilling and Panero 2011; Moraes and Panero 2016; Moreira et al. 2023).

Within Heliantheae, species formed a distinct clade, with *V. encelioides* grouping with *V. alternifolia* (bootstrap 100%), confirming *Verbesina* monophyly. The *Verbesina* clade was sister to *Silphium* and *Echinacea*, while *Bidens*, *Cosmos*, and *Coreopsis* occupied more basal positions. These relationships align with previous plastid‐ and nuclear‐based phylogenetic analyses (Schilling and Panero 2011; Moraes and Panero 2016; Moreira et al. 2023), demonstrating the utility of cp genomes for resolving relationships in taxonomically challenging groups.

## 4 Conclusion

This study reports the first complete cp genome of *Verbesina encelioides* , which shows structural conservation consistent with other Asteraceae species. Comparative analyses revealed high similarity with *V. alternifolia* , with greater variability in intergenic spacers and introns, and several hypervariable regions and SSRs that provide useful markers for population and biogeographical studies. Positive selection was detected at 62 codons across 19 genes, reflecting patterns common in Asteraceae rather than traits linked to invasiveness, suggesting that invasive features are more likely encoded in the nuclear genome. Phylogenetic results confirmed the monophyly of *Verbesina* within Heliantheae.

These genomic resources offer practical value for conservation genetics, evolutionary research, and future assessments of the species' invasive dynamics.

## Author Contributions

**Rushan Yan:** conceptualization (equal), data curation (equal), writing – original draft (equal). **Abdullah:** conceptualization (equal), formal analysis (equal), resources (equal), writing – review and editing (equal). **Jingjing Jia:** data curation (equal), investigation (equal). **Madiha Islam:** investigation (equal), methodology (equal). **Hui Li:** investigation (equal), methodology (equal). **Mengyang Liu:** data curation (equal), formal analysis (equal). **Bartholomew Yir‐erong:** conceptualization (equal), formal analysis (equal), investigation (equal), validation (equal), writing – review and editing (equal). **Xiaoxuan Tian:** conceptualization (equal), visualization (equal), writing – review and editing (equal).

## Funding

This work was supported by the National Natural Science Foundation of China (Grant No. 82474031).

## Conflicts of Interest

The authors declare no conflicts of interest.

## Supporting information


**Table S1:** Analysis of positive selection.

## Data Availability

The assembled chloroplast genome sequence from this study has been deposited in NCBI under the accession number PX334460; the associated raw sequencing data are available under BioProject PRJNA1322372.
